# Lactic Acid Fermentation of Pomegranate Juice as a Tool to Improve Antioxidant Activity

**DOI:** 10.3389/fmicb.2019.01550

**Published:** 2019-07-03

**Authors:** E. Pontonio, M. Montemurro, D. Pinto, B. Marzani, A. Trani, G. Ferrara, A. Mazzeo, M. Gobbetti, C. G. Rizzello

**Affiliations:** ^1^Department of Soil, Plant, and Food Science, University of Bari Aldo Moro, Bari, Italy; ^2^Giuliani S.p.A., Milan, Italy; ^3^CIHEAM-MAIB, Mediterranean Agronomic Institute of Bari, Bari, Italy; ^4^Faculty of Science and Technology, Free University of Bozen-Bolzano, Bolzano, Italy

**Keywords:** pomegranate, lactic acid bacteria, fermentation, antioxidant activity, ellagitannins, Balb 3T3

## Abstract

An increasing consumer demand for pomegranate has been globally observed, mainly thanks to the scientific evidence related to its functional and health-promoting features. Pomegranate fruits from twenty accessions identified in Southeastern Italy were characterized according to morphological and chemical features. Juices extracted from pomegranate fruits were fermented with selected *Lactobacillus plantarum* PU1 and the antioxidant activity investigated. Whey was added to juices to promote the microbial growth. Fermentation led to the increase of the radical scavenging activity (up to 40%) and significant inhibition of the linoleic acid peroxidation. The three fermented juices showing the highest antioxidant activity, and the corresponding unfermented controls, were further characterized. In detail, the cytotoxicity and the protective role toward artificially induced oxidative stress were determined on murine fibroblasts Balb 3T3 through the determination of the viability and the intracellular ROS (reactive oxygen species) scavenging activity (RSA). RSA reached values of *ca.* 70% in fermented juices, being *ca.* 40% higher than the unfermented and control samples. Phenols compounds of the pomegranate juices obtained from accessions “Bitonto Piscina,” “Sanrà nero,” and “Wonderful (reference cultivar) were analyzed through ultrahigh pressure liquid chromatography coupled with mass spectrometry, showing that a marked increase (up to 60%) of the ellagitannins derivatives occurred during fermentation. Sensory analysis showed suitability of the fermented juices to be used as beverage and food ingredient.

## Introduction

Pomegranate (*Punica granatum* L.) is one of the oldest known edible fruits. From Persia, pomegranate native region, its cultivation spread into Asia, North Africa and Europe (Duman et al., 2009; Chandra et al., 2010). The good adaptation to temperate climate favored its wide diffusion throughout the Mediterranean area and the differentiation of several local genotypes (Ferrara et al., 2011, 2014). Indeed, pomegranate species includes a very huge number of domestic, wild and ornamental biotypes showing large variability in phenotypic traits such as fruit size and yield, flowering season, seed-hardness, rind, and aril color. Chemical composition of pomegranate fruits reflects the intra-species biodiversity, since large differences in sugars, fatty acids and polyphenols were reported (Holland et al., 2009; Chandra et al., 2010; Teixeira da Silva et al., 2013).

The rising consumers interest toward health-promoting foods is pressing the industries in re-allocating resources on the development of novel functional foods (Peres et al., 2012; Valero-Cases et al., 2017). This promotes the research on the optimization of new products with high nutritional value to be used as carrier of functional compounds (Valero-Cases et al., 2017).

According to the scientific evidences related to its functional and health-promoting properties (Viuda-Martos et al., 2010), an increasing demand for pomegranate (as fresh fruit or juice) has been observed all over the world (Prospectiva2020, 2015).

The health-promoting properties of pomegranate are mainly related to the abundance of ellagitannins and other polyphenolic compounds (Filannino et al., 2013), and to the correlated antioxidant activity (Gil et al., 2000; Tzulker et al., 2007; Seeram et al., 2008). Indeed, it was reported that the antioxidant activity of pomegranate is higher than red wine, green tea (Gil et al., 2000), grape/cranberry, grapefruit, and orange juice (Roseblat and Aviram, 2006). Besides the antioxidant activity, antiviral (Kotwal, 2007), anti-inflammatory (Giménez-Bastida et al., 2012), and anti-atherosclerotic properties (Aviram et al., 2004) have been attributed to pomegranate compounds. Moreover, the role of the unsaturated fatty acids (including isomers of conjugated linoleic acid) contained in pomegranate seeds in preventing cardiovascular diseases, cancer, asthma and in reducing cholesterol levels has recently been highlighted (Ferrara et al., 2011).

Overall, fruit juices are suitable for the development of functional foods because they are rich in bioactive compounds and meets the consumer’s claims for healthy, tasty, and practical foods (Fonteles and Rodrigues, 2018; Oliveira et al., 2018). Fermentation of plant material *via* lactic acid bacteria has attracted industries consideration to produce fermented foods as alternative source of probiotics (Soccol et al., 2010; Peres et al., 2012). Several studies demonstrated that pomegranate juice fermentation (by lactic acid bacteria, yeasts or filamentous fungi) is a promising tool for further improving its nutritional and functional profile (Trigueros et al., 2014; Gumienna et al., 2016; Cano-Lamadrid et al., 2017). Fermentation with selected lactic acid bacteria allowed to improve antioxidant activity, shelf-life and sensory properties of the pomegranate juice (Filannino et al., 2013). Beside the metabolic conversion of the phenolic compounds by lactic acid bacteria as an efficient mechanism for detoxification (Filannino et al., 2018), the fermentation leads to an intense acidification with a correlated improvement in the release of bioactive compounds. Hence, increasing their bioaccessibility and bioavailability (Hur et al., 2014).

In this scenario, the present study proposes the exploitation of pomegranate juice and lactic acid bacteria fermentation to obtain products with high antioxidant activity to be used in food and beverage industry, to meet consumers requirements. Aiming at investigating the widest local diversity, juices from twenty pomegranate accessions identified in Puglia region (Southeastern Italy), were included in the study and compared to that obtained from Wonderful, the most widespread pomegranate cultivar. Juices were supplemented with whey to promote fermentation, which was carried out with a previously selected *Lactobacillus plantarum* strain as a starter. The antioxidant activity of fermented juices was first evaluated through different *in vitro* assays and then determined on murine fibroblasts cell cultures under artificially induced oxidative stress. The phenolic profile and the sensory characteristics of the fermented juices showing the highest antioxidant activity were also investigated.

## Materials and Methods

### Experimental Design

First, pomegranate fruits collected from twenty local accessions and Wonderful cultivar were characterized for the main morpho-pomological and chemical features. Then, pomegranate juices were supplemented with whey and fermented with *L. plantarum* PU1. The radical scavenging activity of the pomegranate juices was determined, prior to and after fermentation, through DPPH and ABTS assays. The capability of the fermented juices to inhibit the linoleic acid peroxidation during a long-term incubation was also investigated. The cytotoxicity and the protective role of the juices toward artificially induced oxidative stress were determined on murine fibroblasts Balb 3T3 through the determination of the viability and the intracellular ROS (reactive oxygen species) scavenging activity.

Finally, the phenolic and sensory profiles of the pomegranate juices showing the highest potential antioxidant activity were investigated through ultrahigh pressure liquid chromatography coupled with mass spectrometry analysis and through a panel test, respectively.

### Pomegranate Accessions and Fruit Harvesting

Fruits from twenty pomegranate accessions identified in Puglia region (Southeastern Italy) were collected in the harvesting year 2016 (mid-September to mid-October), when greenness generally disappeared from the skin and yellow-pink or red color appeared (an acceptable correlation of the color change with the morpho-pomological and chemical markers of ripening was previously reported by Ferrara et al., 2011). Local accessions and codes are listed in Table 1. The international cultivar Wonderful (coded 21), grown in a private commercial orchard, was included in the study and used as reference.

**Table 1:** Morphological and chemical characteristics of pomegranate skin, arils, and juice.

Accession	Code	Skin thickness (mm)	Total arils weight (g)	100 arils weight (g)	Juice (ml/100 g arils)	Soluble solids (°Brix)	pH	Total titratable acidity (g/L)
Acido torrelonga	1	1.4^a-c^	144.8^b-*d*^	39.9^b-e^	69.2^b-f^	15.6^a-e^	2.84^h^	27.1^*d*^
Bariblu	2	1.1^b,c^	101.0^c-e^	33.2^b-f^	66.0^e,f^	15.8^a-e^	3.41^d-f^	6.5^f,g^
Bitetto dolce	3	1.3^b,c^	155.6^b-*d*^	38.5^b-e^	68.9^c-f^	17.5^a,b^	3.90^a^	3.2^g^
Bitonto piscina	4	1.4^a-c^	36.0^e^	30.3^c-g^	58.1^g^	14.3^c-e^	2.58^i^	54.4^a^
Bitonto T	5	1.3^b,c^	196.7^b^	42.2^b,c^	75.5^a,b^	16.1^a-*d*^	3.51^c-e^	5.3^g^
Campus	6	1.7^a-c^	167.5^b-*d*^	33.7^b-f^	72.3^b-e^	16.9^a-c^	3.49^c-e^	6.4^f,g^
Demarco	7	1.0^c^	146.4^b-*d*^	52.51^a^	75.2^a-c^	14.3^c-e^	3.83^a^	4.9^g^
Dolce conversano	8	1.4^a-c^	201.1^b^	40.5^b-*d*^	73.4^a-*d*^	16.5^a-*d*^	3.60^b,c^	4.2^g^
Dolce corallo	9	2.1^a,b^	130.7^b-*d*^	42.6^b^	70.9^b-e^	16.5^a-*d*^	3.67^b^	3.2^g^
Funno dolce	10	1.4^a-c^	147.3^b-*d*^	36.7^b-f^	70.9^b-e^	17.9^a,b^	3.55^b-*d*^	5.1^g^
Japigia B	11	1.3^b,c^	83.7^d,e^	34.0^b-f^	67.0^d-f^	13.8^d,e^	2.77^h,i^	32.8^c^
Acido capurso	12	1.2^b,c^	279.8^a^	43.7^b^	71.0^b-e^	16.0^a-e^	2.85^h^	26.0^*d*^
Macello triggiano	13	1.6^a-c^	80.5^d,e^	32.3^b-g^	50.0^h^	15.8^a-e^	3.33^e,f^	8.1^f,g^
Melograno foggia	14	1.1^b,c^	176.4^b,c^	26.3^f,g^	69.9^b-e^	17.1^a,b^	3.27^f,g^	7.1^f,g^
Molfetta acido	15	1.9^a-c^	103.2^c-e^	28.1^e-g^	67.0^d-f^	18.2^a^	2.78^h,i^	28.0^*d*^
Mungivacca tardivo	16	1.6^a-c^	111.5^c-e^	27.6^e-g^	65.7^e,f^	14.1^d,e^	2.71^h,i^	33.0^c^
Ottantara	17	2.4^a^	89.7^d,e^	30.3^d-g^	65.4^e,f^	17.5^a,b^	3.15^g^	10.2^f^
Sanrà nero	18	1.8^a-c^	110.7^c-e^	20.8^g^	61.7^f,g^	16.3^a-*d*^	2.65^h,i^	47.6^b^
Modugno	19	1.4^a-c^	94.6^c-e^	39.0^b-e^	67.5^d-f^	13.3^e^	3.35^e,f^	7.0^f,g^
Triggiano	20	1.1^b,c^	106.8^c-e^	26.2^f,g^	77.9^a^	15.5^b-e^	3.30^f,g^	7.5^f,g^

Mean value		1.5 ± 0.3	133.2 ± 29.5	34.9 ± 4.2	68.2 ± 2.4	15.9 ± 0.9	3.22 ± 0.06	16.4 ± 1.4

Wonderful^∗^	21^∗^	1.6^a-c^	297.8^a^	34.1^b-f^	65.3^e,f^	17.6^a,b^	2.86^h^	23.8^e^


*^∗^Wonderful (code 21) was considered as the reference cultivar. Different letters within a column refer to a difference significant at *P* ≤ 0.05, as obtained by the REGWQ test.*

### Pomegranate Fruits and Juices Characterization

Morpho-pomological measurements, arils and seeds characterization, and chemical analyses were carried out on batches of 15 mature fruits for accession, randomly selected on the tree (Martínez et al., 2006; Giancaspro et al., 2017). The following characteristics were determined: fruit weight (g), fruit volume (cm^3^), diameter (mm) and length (mm), sepal number (n.), calyx diameter (mm) and length (mm), skin thickness (mm). Arils were characterized for: total weight (g), 100 arils weight (g), aril maximum width (mm), length (mm) and weight (mg), seed maximum width (mm), length (mm) and weight (mg), juice volume (ml/100 g of arils), total soluble solids (°Brix), pH and titratable acidity (g/l) of juice.

Pomegranate fruits were analyzed within 2 h from harvesting. Skins and arils were separated from each of the 15 fruits collected from local accessions and Wonderful. Juice was prepared by squeezing the arils through a metal sieve. The juice extraction yield was *ca.* 65–70% (ml/100 g of arils). Juices were sterilized by filtration on 0.22 μm membrane filters (Millipore Corporation, Bedford, MA 01730). The soluble solids (°Brix) were determined through a digital refractometer model HI96814 (Hanna Instruments, RI, United States). Total titratable acidity (TTA) was determined by using a semi-automatic titrator (PH-Burette 24, Crison, Spain) on 5 ml of juice added with 45 ml of distilled water. Titration was carried out with 0.1 N NaOH to a final pH of 8.1. TTA was expressed as g of citric acid/1000 ml juice. Aliquots of juices were frozen at -20°C until fermentation and further analyses. Measurements were made in triplicates for each juice.

### Starter and Culture Conditions

*Lactobacillus plantarum* PU1, belonging to the Culture Collection of the Department of Soil, Plant and Food Science (University of Bari Aldo Moro, Italy) was used as starter for pomegranate juice fermentation. The strain was previously identified by partial 16S rRNA and recA sequencing. *L. plantarum* PU1 was cultivated on De Man, Rogosa and Sharpe (MRS, Oxoid, Basingstoke, Hampshire, United Kingdom) broth supplemented with cycloheximide (Sigma-Aldrich, St. Louis, MO, United States) (0.1 g/l) at 30°C. When used for fermentation, *L. plantarum* PU1 was cultivated until the late exponential phase of growth was reached (*ca.* 12 h). Then, cells were harvested by centrifugation (10,000 ×*g*, 10 min, 4°C) and washed twice with sterile potassium phosphate buffer (50 mM, pH 7.0). Cells were re-suspended in juices at the optical density (OD_620_) of 2.5, corresponding to the cell density of *ca.* 9.0 Log cfu/ml. Enumeration of lactic acid bacteria was carried out by plating onto MRS (Oxoid) agar at 30°C for 48 h.

### Pomegranate Juice Fermentation

Pomegranate juices were added (1:1, v/v) with a reconstituted whey and mixed using a Stomacher 400 lab blender (Seward Medical, London) for 10 min. In particular, reconstituted whey contained 10% w/v whey powder (Sigma Aldrich, 9% protein content). The mixture (Pj) was centrifuged (6,000 ×*g*, 10 min, 4°C) and kept at 4°C for 2 h, prior inoculation. Then, Pj were inoculated with the starter and fermented (FPj). The initial cell density of the starter was *ca.* 2 × 10^7^ cfu/ml. Fermentation was carried out at 30°C for 24 h, under stirring conditions (120 rpm). FPj were centrifuged (10,000 ×*g* for 20 min) at 4°C, and the supernatants were used for analyses. The growth of *L. plantarum* PU1 in FPj was monitored through enumeration on MRS (Oxoid) agar medium. The values of pH were determined by a pH-meter (Model 507, Crison, Milan, Italy) with a food penetration probe.

### *In* *vitro* Antioxidant Activity

#### Radical Scavenging Activity on 1,1-Diphenyl-2-Picrylhydrazyl (DPPH)

The scavenging activity of Pj and FPj on DPPH free radical was measured according to the method of Shimada et al. (1992) with some modifications. Two milliliters of each Pj and FPj were added to 2 ml of 0.1 mM DPPH dissolved in 95% ethanol. The mixture was shaken and incubated at room temperature. The absorbance (517 nm) was measured after 10 min and used for the calculation of the scavenged DPPH (Rizzello et al., 2010). The scavenging activity was expressed as follows: DPPH scavenging activity (%) = [(blank absorbance - sample absorbance) / blank absorbance] × 100. The whey solution used for pomegranate juice supplementation was also analyzed (prior and after fermentation with *L. plantarum* PU1). Butylated hydroxytoluene (BHT, 75 ppm) was used as the antioxidant reference (Rizzello et al., 2010).

#### Scavenging Activity on 2,2′-Azino-di-[3-Ethylbenzthiazoline Sulphonate] (ABTS^⋅+^)

The scavenging activity of Pj or FPj toward radical cation ABTS^⋅+^ was determined through the Antioxidant Assay Kit CS0790 (Sigma Chemical Co.), following the manufacturer’s instruction. The antioxidant compound Trolox (6-hydroxy 2,5,7,8-tetramethylchroman-2-carboxylic acid) was used for obtaining the calibration curve. The antioxidant activity of the juices was reported as mM Trolox equivalent (eq.).

#### Inhibition of Linoleic Acid Peroxidation

The capability of Pj and FPj to inhibit the linoleic acid peroxidation was measured according to the method of Osawa and Namiki (1985) with some modifications (Rizzello et al., 2013). Linoleic acid (50 mM, in ethanol) was added to Pj and FPj (1:1 ratio), and the mixtures incubated in the dark at 60°C, using glass tubes sealed with silicone rubber caps, for 8 days.

The oxidation level was determined during incubation as previously reported by Mitsuta et al. (1996), by measuring the ferric thiocyanate. In details, 100 μl of each sample mixture was added to 4.7 ml of 75% (v/v) ethanol, 100 μl of 30% (w/v) ammonium thiocyanate, and 100 μl 20 mM ferrous chloride in 1 M HCl.

Absorbance at 500 nm was measured after 3 min of incubation at room temperature. A positive control, corresponding to a BHT solution (1 mg/ml), and a negative control (without antioxidant) were included in the analysis. The inhibition of linoleic acid peroxidation (%) was calculated as follows: [(negative control absorbance – sample absorbance)/negative control absorbance] × 100.

### Total Phenols Concentration

Total phenols were determined on the freeze-dried ME of Pj and FPj with the Folin–Ciocalteu method as described by Slinkard and Singleton (1997). Five grams of each sample were mixed with 50 ml of 80% methanol to get ME. The mixture was purged with nitrogen stream for 30 min, under stirring condition, and centrifuged at 4600 ×*g* for 20 min. MEs were transferred into test tubes, purged with nitrogen stream and stored at *ca.* 4°C before analysis. The concentration was expressed as gallic acid equivalent.

### Cytotoxicity of the Pomegranate Juices

Balb 3T3 murine fibroblasts (clone CCL-163^TM^) were provided by ATCC Culture Collection (Middlesex, United Kingdom). Fibroblasts were cultivated in Dulbecco’s Modified Eagle Medium (DMEM) added with 10% (w/v) calf bovine serum (CBS), renewed every 2 days. A mixture penicillin (10,000 U/ml)/streptomycin (10,000 U/ml) and a non-essential amino acid solution (NEAA) were both added at 1%. Cell cultures were grown under humidified atmosphere (5% CO_2_, 37°C).

Cell viability was determined by the MTT [3-(4,5-dimethyl-2-yl)-2,5-diphenyltetrazolium bromide] assay (Mosmann, 1983), based on the capability of succinate dehydrogenase to convert 3-(4,5-dimethylthiazol-2-yl)-2,5-diphenyltetrazolium bromide into formazan crystals. Viability assays was performed on cell cultures after four passages on the DMEM. In detail, cells were inoculated into 96-well plates (Becton Dickinson France S.A., Meylan Cedex, France) at the density of 5 × 10^4^ cells/well. Incubation lasted 24 h necessary to obtain approximately 80% confluence.

Aiming at determining the non-cytotoxic concentration, cells were incubated in presence of freeze-dried Pj and FPj at the final concentration of 0.1, 1, 5, 10, 50, and 100 mg/ml Basal medium without the Pj and FPj was used for the control. Viability was determined, for each concentration, after 24, 48, and 72 h of incubation.

One hundred microliters of MTT solution (0.5 mg/ml final concentration) in DMEM, were added to each well at the end of the incubation, after removing the medium. After 3 h of incubation (5% CO_2_, 37°C) in the dark, purple formazan products were dissolved by adding 100 μl of dimethyl sulfoxide (DMSO). Then, the reaction mixture was incubated in stirring conditions for 15 min at room temperature, in the dark. A microplate reader (BioTek Instruments Inc., Bad Friedrichshall, Germany) was used to measure absorbance at 520 nm.

### Protective Effect on Balb 3T3 Cells Subjected to Artificially Induced Oxidative Stress

The viability of H_2_O_2_-stressed Balb 3T3 cells was also determined by the MTT assay (Coda et al., 2012; Rizzello et al., 2013). Before inducing the oxidative stress, cells were incubated for 16 h in presence of the freeze-dried Pj and FPj at the final concentration of 1, 5, and 10 mg/ml. DMEM supplemented with 2.5% CBS, 1% penicillin (10,000 U/ml), streptomycin (10,000 U/ml), and 1% non-essential amino acids solution (NEAA) was the basal medium. The positive control was represented by α-tocopherol (250 and 500 μg/ml). After incubation, wells were washed, and the cells incubated for 2 h in 400 μM H_2_O_2_ (100 μl/well) dissolved in DMEM. Cells not incubated with Pj or FPj and cells not subjected to the oxidative stress were used as controls.

The viability of H_2_O_2_-stressed Balb 3T3 cells after incubation was determined by the MTT assay (Coda et al., 2012; Rizzello et al., 2013). Data were calculated as the percentage of viable cells compared to the cells not subjected to the oxidative stress. Data are expressed as the mean of three independent experiments.

### Intracellular Reactive Oxygen Species (ROS) Determination

The 2′,7′-dichlorofluorescein diacetate (DCFH-DA) assay (Tobi et al., 2000) was used to investigate the reactive oxygen species (ROS) generation in murine fibroblasts (Balb 3T3). The use of DCFH-DA is based on the capability of the non-polar non-ionic DCFH-DA to cross cell membranes, where it is enzymatically hydrolyzed by intracellular esterases to non-fluorescent DCFH. In the presence of ROS, DCFH is rapidly oxidized to highly fluorescent 2′,7′-dichlorofluorescein (DCF).

Cells were cultivated in DMEM supplemented with 2.5% CBS, and incubated with freeze-dried Pj and FPj (1, 5, and 10 mg/ml) for 16 h as described above. At the end of the incubation cells were washed twice with phosphate buffer saline (PBS). A DCFH-DA stock solution (40 mM), previously obtained in DMSO (>99.5%), was diluted (0.25%) with basal medium. One hundred microliters of such DCFH-DA solution (final concentration 100 μM) were added to each well of the 96-wells plate, incubated for 30 min at 37°C, in the dark. Cells were washed with PBS and subjected to oxidative stress as described above. H_2_O_2_-stressed cells not previously treated with Pj and FPj were also analyzed.

After the H_2_O_2_ treatment, cells were washed twice and added with a lysis buffer (Cell Lytic M, Sigma Chemical Co.) and 1% protease inhibitor cocktail (Sigma Chemical Co.). A Fluoroskan Ascent FL Microplate Fluorescence Reader (Thermo Fisher Scientific) was used to measure the Fluorescent 2′,7′-dichlorofluorescein (DCF). Excitation and emission wavelengths were 485 and 538 nm, respectively. Data are the mean of three independent experiments and corresponded to the percentage of radical scavenging activity (RSA) compared to the control (H_2_O_2_-stressed cells not treated with antioxidants).

### Phenols Characterization

Phenols compounds were analyzed through UHPLC-PAD-HESI-MS/MS (ultrahigh-pressure liquid chromatography coupled with MS/MS Mass Spectroscopy) analysis. Before analysis, Pj and FPj were diluted 1:1 (v/v) with formic acid 0.1% and filtered at 0.2 μm pore size using regenerated cellulose syringe adapter filters. The chromatographic separation of the phenols was performed using an ultrahigh-pressure liquid chromatograph (UHPLC) Ultimate 3000 system (Dionex Thermo Fisher Scientific, Rodano, Italy) equipped with LPG-3400RS pump, WPS-3000 autosampler, TCC-3000 column oven, and a Photodiode Array Detector PDA 3000. System was coupled to a Triple Quadrupole Mass Spectrometer TSQ Quantum Access Max (Thermo Fisher Scientific) through a Heated Electrospray Ionization HESI-II probe.

The UHPLC analysis was carried out at constant flow (0.3 ml/min) using a Hypersyl Gold column (Thermo Fisher Scientific) having 100 mm length, 2.1 mm internal diameter, and 3 μm-particle size. Elution was performed by a mobile phase consisting of solvent A, formic acid 0.1% in water, and solvent B, formic acid 0.1% in acetonitrile. A linear gradient from 2 to 25% solvent B in 20 min was used. The UV/VIS spectrum was acquired from 220 to 600 nm.

The mass detector was tuned using quercetin 1 ppm in continuous infusion. The mass detector operating conditions were: capillary temperature 280°C and ion transfer tube temperature 320°C. Acquisition of 4 mass events in series in full mass scan (from 250 to 800 atomic mass units) was carried out, followed by MS/MS transition by using the three main signals of the first event. MS/MS experiments were carried out by using a standardized 35 eV collision induced dissociation energy. All the compounds were identified according to the mass spectral characteristics (mass spectra, accurate mass, and characteristic fragmentation).

### Sensory Analysis

Sensory analysis of pomegranate juices was carried out by 10 trained panelists (5 male and 5 female, mean age: 35 years, range: 22–50 years). Anise, astringent, berry, fermented, floral, fruity, grape, pungent, sour, sweet, vinegar, wine-like, and molasses were considered as sensory attributes using a scale from 0 to 10, as previously described by Di Cagno et al. (2017). Moreover, color and browning attributes were also included (Filannino et al., 2013). The sensory attributes were discussed with the assessors during an introductory sensory training session. Samples were randomly coded and served (20 ml) at room temperature, together with non-salted table biscuits and water. Samples were evaluated in three replicate sessions using two replicates for each condition. According to the IFST Guidelines for Ethical and Professional Practices for the Sensory Analysis of Foods, assessors gave informed consent to tests and could withdraw from the panel at any time, without penalty or having to give a reason.

### Statistical Analysis

For the data regarding pomegranate fruit characterization, variance assumptions were verified through Levene’s test for homogeneity of variance and Lillefors and Shapiro–Wilk tests for normal distribution. Analysis of variance (ANOVA) was carried out with the software XLSTAT-Pro (Addinsoft, Paris, France) at the P level of 0.05. The means of the data corresponding to different treatments were statistically separated by the REGWQ test. Biochemical data for juices were treated by one-way ANOVA; pair-comparison of treatment means was obtained by the Tukey’s procedure (*P* < 0.05), by using Statistica for Windows (version 12.5). Student’s *t*-test was applied to MTT assay results (GraphPAD 6.0 for Windows).

## Results

### Pomegranate Fruits Characterization

The weight of pomegranate fruits of the local accessions ranged from 115.5 ± 16.1 to 548.0 ± 57.3 g (Supplementary Table S1) whit mean value of 252.9 ± 42.6 g being significantly (*P* < 0.05) lower than the weight of Wonderful cultivar (616.1 ± 31.9 g). Moreover, only 8 accessions had fruits with weight exceeding the mean value. Sepal number mean value was 6.3 ± 0.5, with accession 12 having the highest value (Supplementary Table S1). The diameter of the calyx ranged from 12.3 ± 1.1 (accession 7) to 27.4 ± 1.1 (accession 1), while its length showed a small variability (from 15.5 ± 2.1 to 10.0 ± 2.6 for accessions 3 and 14, respectively). The mean value for skin thickness was 1.5 ± 0.3 mm, except for accessions 9 and 17 (over 2 mm) (Table 1). With the only exception of accession 12 which had the highest total aril weight (279.8 ± 67.2 g), all accessions were characterized by values lower than 200 g and anyway lower than that of Wonderful (Table 1). The fresh weigh of 100 arils was 34.9 ± 4.2 g, similarly to Wonderful (34.1 ± 3.3 g).

The juice yield, with a mean value of 68.2 ± 2.4 ml/100g arils, showed significant differences among samples (Table 1) and the highest value (77.9 ± 0.9 ml) was observed for the accession 20. The highest and lowest sugar concentrations were found in accessions 15 and 19, respectively. TTA showed a wide variability, from *ca.* 3.2 (accessions 3 and 9) to 54.4 ± 2.7 g/l (accession 4). Six accessions had a TTA higher than 25 g/l (Table 1). The pH mean value was 3.22 ± 0.10.

The aril and seed size values were the highest in accessions 9, 12, 19, and 20, whereas the lowest values were observed in two wild accessions (4 and 18) having very dark skin (Table 2). The mean arils weight for the accessions considered in the study was similar to that of Wonderful (340 ± 57 vs. 327 ± 58 mg), however, significant differences were found among local accessions (from 208 ± 38 to 505 ± 103 mg). Similar trend was found for 100 arils weight (values ranged from 20.8 ± 5.4 to 52.5 ± 1.2 g). Moreover, small variability was found for seed weight values.

**Table 2:** Morphological characteristics of pomegranate aril and seed.

Accessions	Aril length (mm)	Aril diameter (mm)	Aril weight (mg)	Seed length (mm)	Seed diameter (mm)	Seed weight (mg)
1	9.9^c-f^	7.3^a-e^	347^e-g^	6.6^b,c^	3.1^a^	19^b,c^
2	9.9^c-f^	7.1^b-e^	316^f-h^	6.5^c^	2.8^b-*d*^	22^b,c^
3	10.1^b-*d*^	7.0^b-e^	361^c-f^	6.3^c,*d*^	2.7^b-*d*^	25^b^
4	7.1^h^	5.3^h^	306^g,h^	5.2^f^	2.3^e^	20^b,c^
5	10.8^b^	6.7^d-g^	387^c-e^	7.0^a,b^	2.6^*d*^	11^c^
6	10.0^c-e^	7.1^b-e^	340^f,g^	6.0^d,e^	2.6^d,e^	18^b,c^
7	9.6^e,f^	6.1^g^	254^i^	6.6^b,c^	2.8^a-*d*^	22^b,c^
8	10.7^b^	7.9^a^	400^c^	6.3^c,*d*^	2.7^c,*d*^	18^b,c^
9	11.3^a^	7.9^a^	441^b^	6.7^b,c^	2.7^b-*d*^	21^b,c^
10	10.1^b-e^	7.4^a-*d*^	354^d-g^	6.4^c,*d*^	2.7^c,*d*^	21^b,c^
11	9.9 ^c-f^	6.7^d-g^	315^f-h^	6.2^c-e^	2.6^d,e^	21^b,c^
12	10.6^b,c^	7.7^a,b^	404^c^	6.4^c,*d*^	3.1^a^	24^b,c^
13	10.4^b-*d*^	6.8^c-g^	342^f,g^	6.5^c^	2.9^a-*d*^	24^b,c^
14	9.3^f^	6.2^f,g^	259^i^	6.4^c,*d*^	2.6^d,e^	17^b,c^
15	9.2^f^	6.9^c-f^	271^h,i^	5.7^e^	2.7^b-*d*^	19^b,c^
16	9.7^d-f^	7.1^b-e^	317^f-h^	6.4^c,*d*^	3.0^a-c^	22^b,c^
17	9.9^c-f^	6.6^e,g^	283^h,i^	6.5^c,*d*^	2.9^a-*d*^	17^b,c^
18	8.5^g^	6.1^g^	208^j^	6.2^c-e^	3.1^a,b^	26^b^
19	10.8^b^	7.5^a,c^	394^c,*d*^	7.0^a,b^	3.1^a^	23^b,c^
20	11.7^a^	7.9^a^	505^a^	7.1^a^	3.1^a^	26^a^

Mean value	10.0 ± 0.9	7.0 ± 0.9	340 ± 57	6.4 ± 0.5	2.8 ± 0.4	21 ± 3

21^∗^	9.9^c-f^	6.4^a-g^	327^e-h^	6.7^b,c^	2.5^a-e^	21^b,c^


*^∗^Wonderful (code 21) was considered as the reference cultivar. Different letters within a column refer to a difference significant at *P* ≤ 0.05, as obtained by the REGWQ test.*

### Microbial Growth and Acidification Capacity

The fresh pomegranate juice allowed a very poor growth of *L. plantarum* PU1. Contrarily, when whey powder solution was added to the juice (Pj) the cell density of the starter at 24 h incubation increased from 0.5 to 1.5 logarithmic cycles (7.6 ± 0.2 – 8.8 ± 0.4 Log cfu/ml), remaining stable up to 48 h of incubation. All further experiments were carried out on Pj supplemented with whey solution (10%, w/v) and fermented for 24 h.

Initial values of pH in Pj, after the addition of the whey solution, ranged from 3.04 ± 0.12 to 5.56 ± 0.21 and decreased to 3.04 ± 0.17 – 4.53 ± 0.16, after 24 h.

Under the above optimal conditions, the concentration of lactic and acetic acids after the fermentation varied from 2.2 ± 0.04 (FPj4) to 40.5 ± 0.61 (FPj17) mmol/l and from 0.7 ± 0.06 (FPj4) to 3.3 ± 0.05 (FPj3) mmol/l, respectively. Median value of 0.4 ± 0.002 mmol/l of lactic acid was found in Pj. Acetic acid was not detectable in any Pj sample.

### Antioxidant Activity and Total Phenols Concentration

**FIGURE 1:**
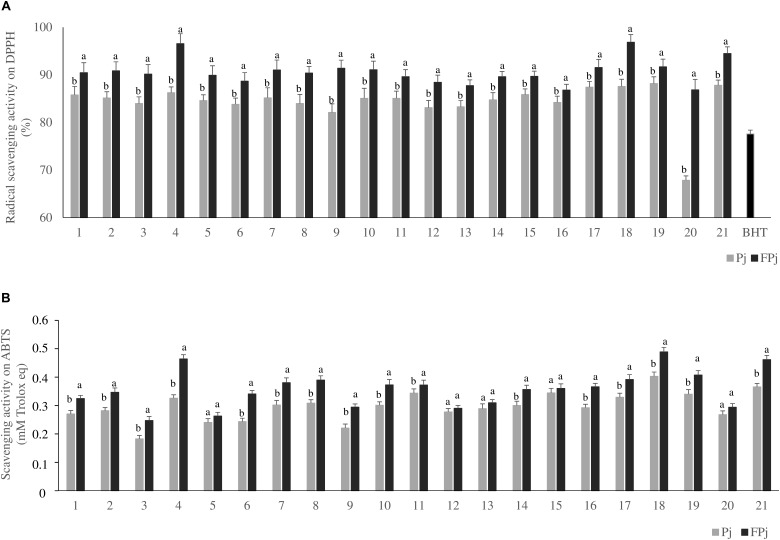
Radical scavenging activity on DPPH (panel **A**) and ABTS (panel **B**) of the pomegranate juices prior (Pj) and after (FPj) fermentation with *Lactobacillus plantarum* PU1. Error bars are shown. ^a,b^Different superscript letters referred to significant (*P* < 0.05) difference between antioxidant activity of the juice prior and after fermentation.

Pomegranate juices prior (Pj) and after the fermentation for 24 h at 30°C with *L. plantarum* PU1 (FPj) were assayed for the antioxidant activity by three different *in vitro* methods (Figure 1A,B).

First, the radical scavenging activity on stable DPPH radical was determined. Aiming at excluding the contribution of the whey solution to the antioxidant activity of the pomegranate juices, its radical scavenging activity on DPPH was previously determined. It corresponded to 7.3 ± 0.7 and 7.6 ± 1.2% prior to and after fermentation with *L. plantarum* PU1, respectively. Under the assay conditions, the 100% of activity corresponded to the complete scavenging of DPPH radical (50 μM final concentration) after 10 min of incubation with the antioxidant compounds. Overall, with the only exception of the sample Pj20 (*ca.* 67.8 ± 0.9%), the radical scavenging activity toward the stable radical DPPH of Pj was significantly higher than that of BHT (positive control), corresponding to 77.6 ± 0.8%. Compared to values observed for Pj, fermentation significantly (*P* < 0.05) increased the radical scavenging activity in all the samples. All the FPj had values higher than *ca*.86% with FPj4 and FPj18 having the highest value (*ca.* 96%) (Figure 1A).

**FIGURE 2:**
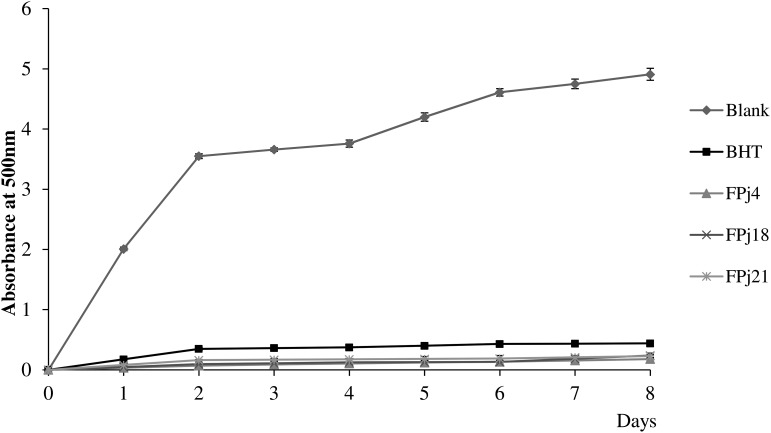
Kinetics of the linoleic acid peroxidation monitored during 8 days of incubation at 60°C in samples containing the fermented pomegranate juices (FPj) and BHT (1 mg/ml). A blank, corresponding to the reaction mixture without pomegranate juice addition, was included in the analysis.

Then, the scavenging activity of Pj and FPj toward radical cation ABTS was determined (Ragaee et al., 2006). The ABTS scavenging activity of the Pj was in the range 0.18 ± 0.01 – 0.40 ± 0.01 mM Trolox eq. (Figure 1B). FPj were characterized by a higher antioxidant activity with values ranging from 0.26 ± 0.01 to 0.49 ± 0.05 mM Trolox eq (Figure 1B). Overall, the ABTS scavenging activity significantly (*P* < 0.05) increased during the fermentation in sixteen out of 21 samples. Moreover, FPj4, FPj18, and FPj21 had values higher than 0.45 mM Trolox eq. The inhibition of linoleic acid peroxidation was also determined as an index of the long-term antioxidant activity. Lipid peroxidation is thought to proceed via radical mediated abstraction of hydrogen atoms from methylene carbons in polyunsaturated fatty acids (Qian et al., 2008). The peroxidation inhibition caused by the FPj at the end of the 8 days of incubation was significantly (*P* < 0.05) higher than that of BHT (82.5 ± 0.5%). The highest inhibitory activity was observed for FPj18 (96.34 ± 0.08%) and FPj21 (95.18 ± 0.05%), followed by the slightly but significantly (*P* < 0.05) lower value observed for Fpj4 (91.04 ± 0.04%). The kinetics of the linoleic acid peroxidation in samples containing BHT, Fpj4, Fpj18, and Fpj21 are reported in Figure 2.

To quantify the total phenols concentration, Folin–Ciocalteu method was applied. All samples had a concentration of total phenols higher than 150 mg/l, with FPj4, FPj18, and FPj21 having the highest concentrations (300.9 ± 3.6, 364.4 ± 5.9, and 374.9 ± 4.2mg/l, respectively). No significant (*P* > 0.05) differences were found between FPj18 and FPj21 phenols concentration.

Based on the above results, FPj4, FPj18 and FPj21 showed the highest antioxidant activity and therefore further characterized by means of the antioxidant activity on biological system and phenolic profiles through UHPLC-PAD-HESI-MS/MS.

### Cytotoxicity

**FIGURE 3:**
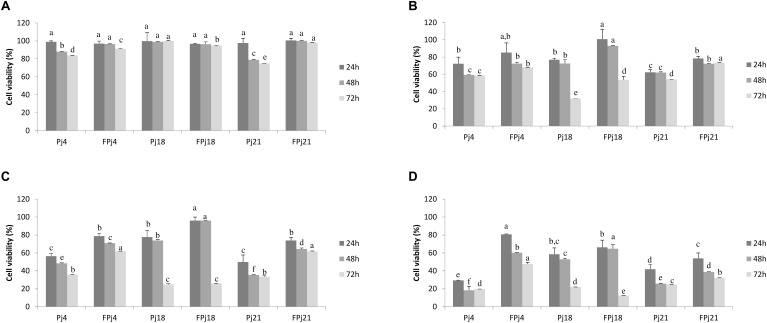
Cell viability of mouse fibroblasts treated with freeze-dried pomegranate juices at different concentrations [0.1 mg/ml, panel **(A)**; 1 mg/ml, panel **(B)**; 5 mg/ml, panel **(C)**; and 10 mg/ml, panel **(D)**]. Viability was determined after 24 (dark gray), 48 (gray), and 72 (light gray) h from the end of the treatment. Data are the means of three independent experiments twice analyzed. Error bars are shown. ^a-f^Values obtained at the same time with different superscript letters differ significantly (*P* < 0.05).

Cytotoxicity of the freeze-dried Pj and FPj was determined on murine fibroblasts Balb3T3. The fibroblast viability after the exposure to pomegranate juices was determined through the MTT assay (Figure 3A–D). Overall, the cytotoxic effect increased according to the concentration of the extract used, indeed values of 50 and 100 mg/ml led to the death of the cells regardless the type of pomegranate juice (Figure 3A–D). FPj showed lower cytotoxicity than corresponding Pj. Samples at concentration of 0.1 mg/ml did not affect cell viability, with the only exception of Pj21 that caused a significant decrease after 48 h of incubation (Figure 3A). Concentration of 1 mg/ml caused cytotoxic effect since 24 h of incubation (Figure 3B), with the only exception of FPj18, whose effect became evident only after 48 h. Although a further decrease of the cell viability occurred increasing samples concentration to 5 mg/ml (Figure 3C), FPj18 still did not cause any significant effect before 48 h of incubation. Viability strongly decreased when 10 mg/ml were used regardless the sample (Figure 3D), with Pj4 and Pj21 causing the highest cytotoxic effect.

### Protective Effect Toward Oxidative-Induced Stress in Balb3T3 Cells

Balb 3T3 cells, cultivated in presence of pomegranate juice, were subjected to oxidative stress by a treatment with hydrogen peroxide. Freeze-dried Pj and FPj were assayed at concentrations lower than 10 mg/ml to avoid cytotoxic effects on fibroblasts.

**FIGURE 4:**
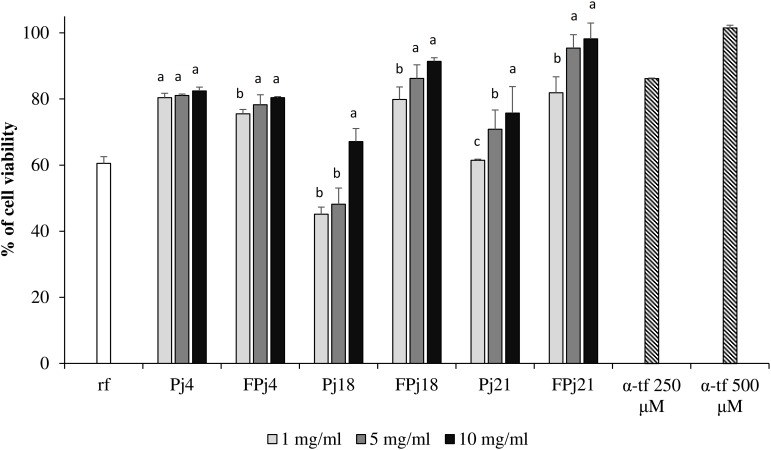
Protective effect of different concentrations (1–10 mg/ml) of freeze-dried pomegranate juices and α-tocopherol (α-tp; 250 and 500 μM) on the cell viability of mouse fibroblasts subjected to oxidative stress induced by hydroxide peroxide. The viability of H_2_O_2_-stressed cells incubated without antioxidant compounds (reference, rf) was also included. Data are the means of three independent experiments twice analyzed. Error bars are shown. ^a-c^Values obtained at the same concentration with different superscript letters differ significantly (*P* < 0.05).

Cell viability after oxidative stress was 60.55 ± 2.03% for the negative control (no antioxidants added), while treatments with α-tf, Pj and FPj significantly (*P* < 0.05) increased cell survival (Figure 4). For all the concentrations tested, the protective effect of FPj was significantly (*P* < 0.05) higher than that of Pj, with the only exception of the Pj/FPj4.

In detail, treatments with 5 and 10 mg/ml of FPj18 and FPj21 corresponded to cell viability (86.24 ± 2.09 and 91.36 ± 1.14%, and 95.40 ± 4.04 and 98.18 ± 4.78%, respectively) higher than that induced by α-tf 250 μM (86.17 ± 1.02%). FPj21 effect was similar (*P* > 0.05) to that of 500 μM α-tf.

### Intracellular Reactive Oxygen Species (ROS) Determination

**FIGURE 5:**
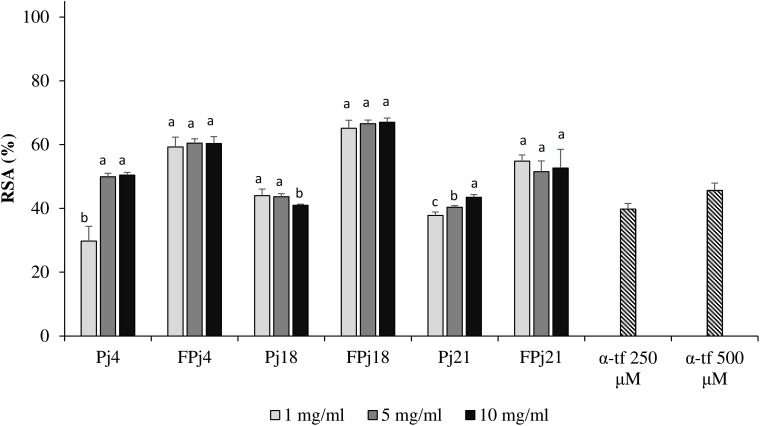
Effect of different concentrations (1–10 mg/ml) of freeze-dried pomegranate juices and α-tocopherol (α-tp; 250 and 500 μM) on the radical scavenging activity (RSA) of mouse fibroblasts subjected to oxidative stress induced by hydroxide peroxide. The RSA of the H_2_O_2_-stressed cells incubated without antioxidant compounds (reference, rf) was also included. Data are the means of three independent experiments twice analyzed. Error bars are shown. ^a-c^Values obtained at the same concentration with different superscript letters differ significantly (*P* < 0.05).

To confirm the antioxidant potential of pomegranate juice against oxidative stress, murine fibroblasts were grown in presence of Pj, FPj, or α-tocopherol, incubated with DCFH-DA, and then treated with hydrogen peroxide. DCFH-DA acted as the probe to monitor the generation of intracellular ROS by flow cytometry. The α-tocopherol treatment induced a RSA of *ca.* 40% (Figure 5). Similar values were observed for cells treated with 5–10 mg/ml of the Pj, except for Pj4 which led to a significantly higher RSA (*ca.* 50%). Overall, the highest activity was found for FPj and it was not significantly different among the three concentrations used. When the pre-treatment was carried out with FPj18, RSA reached the highest values of *ca.* 70%.

### Phenolic Profiles

The phenolic profiles of FPj4 and 18 were investigated through UHPLC-PAD-HESI-MS/MS and compared to those of the corresponding unfermented Pj. Profiles of the unfermented and fermented juices obtained from the commercial cultivar Wonderful (Pj21 and FPj, respectively) were also analyzed. The analysis allowed the identification of the most polar anthocyanin pomegranate pigments (Fischer et al., 2011) such as delphinidin 3,5-diglucoside, which eluted first, followed by cyanidin 3,5-diglucoside, delphinidin 3-glucoside, a cyanidin–pentoside–hexoside, cyanidin 3-glucoside, and pelargonidin 3-glucoside (Table 3). Phenolic profiles of the juices prior fermentation were similar, nevertheless, Pj21 showed the highest peak area ratio of delphinidin 3,5-diglucoside, hexahydroxydiphenoyl-hexoside (HHDP-hex), ellagic acid dihexoside, pelargonidin 3-glucoside (Table 3).

**Table 3:** Phenols compounds identified in pomegranate juices: retention time, peak area, mass spectra characteristics.

Assignment	t_R_ (min)	Peak area^∗^ (%)						UHPLC-PAD λmax (nm)	[M-H]^-^ m/z	MS/MS fragments m/z (% base peak)	References
		Pj4	FPj4	Pj18	FPj18	Pj21	FPj21				
**Delphinidin 3,5-diglucoside**	4.96	9.712^c^	9.743^c^	9.461^*d*^	9.611^c^	10.263^b^	11.536^a^	280, 330, 524	625	301 (100) 284 (60) 241 (55)	Fischer et al., 2011; Mena et al., 2012; Brighenti et al., 2017
**HHDP-hexoside ^(1)^**	5.09	3.034^f^	4.634^*d*^	3.627^e^	5.756^c^	6.752^b^	8.553^a^	280, 350, 524	480	124 (100) 197 (40) 165 (30)	Fischer et al., 2011
**Ellagic acid dihexoside ^(1)^**	5.96	9.045^*d*^	10.621^c^	8.934^*d*^	13.398^a^	10.224^c^	11.875^b^	275, 331, 396, 519	626	301 (100) 462 (60)	Fischer et al., 2011
**Cyanidin 3,5-diglucoside**	7.11	8.023^b^	8.222^b^	8.865^a^	8.960^a^	8.003^b^	7.943^b^	513, 277	608	284 (100) 446 (20)	Fischer et al., 2011; Mena et al., 2012; Brighenti et al., 2017
**Delphinidin 3-glucoside**	7.58	42.122^a^	37.221^b^	40.059^a^	36.424^b^	38.321^b^	35.076^c^	521, 274, 343	463	300 (100)	Fischer et al., 2011; Mena et al., 2012
**Cyanidin 3-glucoside**	7.84	9.021^b^	9.201^b^	9.937^a^	9.820^a^	8.945^b^	9.229^b^	515, 280, 330	449	287 (100)	Fischer et al., 2011; Mena et al., 2012; Brighenti et al., 2017
**Cyanidin–pentoside–hexoside**	8.09	4.865^a^	5.042^a^	4.105^b^	4.260^b^	4.055^b^	4.035^b^	275, 520	581	449 (100), 287 (77), 419 (30)	Fischer et al., 2011
**Pelargonidin 3-glucoside**	8.78	5.025^c^	5.253^c^	5.546^b^	4.979^c^	6.243^a^	6.064^a^	501, 271, 330, 428	433	271 (100)	Fischer et al., 2011; Mena et al., 2012; Brighenti et al., 2017
**Ellagic acid deoxyhexoside ^(1)^**	11.96	1.522^*d*^	2.076^c^	2.249^b^	3.141^a^	1.822^c^	2.336^b^	273	447	300 (100), 301 (80), 302 (17)	Mena et al., 2012; Brighenti et al., 2017


*t_*R*_, Retention time. ^∗^Peak area (%) was calculated as follows: (peak area/total peak area) × 100. λ520 nm-UHPLC chromatograms were considered for calculation, except for non-anthocyanin phenolic compounds (1), whose area ratios referred to λ280 nm-UHPLC chromatograms. ^*a*-*f*^Values in the same raw with different superscript letters differ significantly (*P* < 0.05).*

The ratio of the peak area of different compounds on the total peak area varied during fermentation (Table 3). The most relevant changes during the fermentation were observed for delphinidin 3-glucoside that decreased in all the samples, and for HHDP-hex, ellagic acid dihexoside, and ellagic acid deoxyhexoside, whose peak area ratios were significantly (*P* < 0.05) higher in FPj compared to corresponding Pj. In detail, increases of ca. 60, 50, and 40% were, respectively found for HHDP-hex, ellagic acid dihexoside, and ellagic acid deoxyhexoside in FPj18 compared to Pj18 (Table 3). Compared to the other fermented juices, FPj18 was characterized by the highest increases and peak area of such compounds.

### Sensory Analysis

The overall acceptability of all the pomegranate juices used in this study was assessed during preliminary sensory training sessions (data not shown), while the sensory descriptive analysis was carried out by the trained panel on the Pj and FPj with the highest antioxidant activity.

**FIGURE 6:**
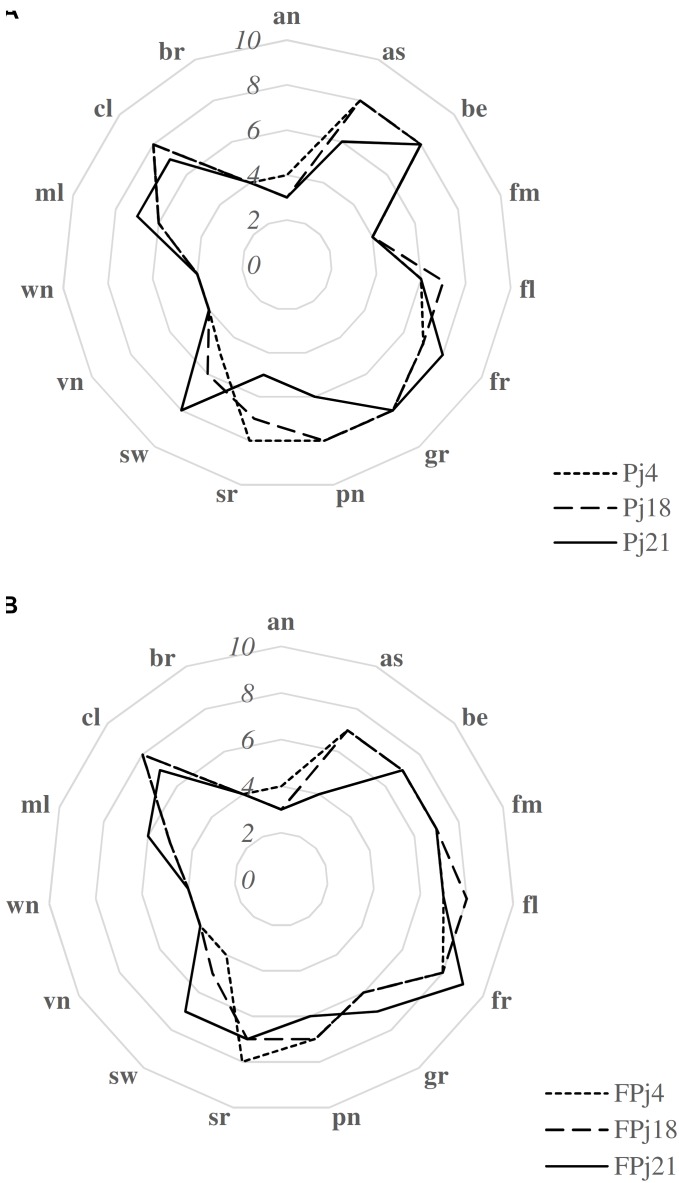
Sensory analysis of the unfermented [Pj4, Pj18, Pj21, panel **(A)**] and fermented [FPj4, FPj18, Fpj21, panel **(B)**] pomegranate juices. Attributes used in the sensory descriptive analysis (Filannino et al., 2013; Di Cagno et al., 2017) are: anise (an), astringent (as), berry (be), fermented (fm), floral (fl), fruity (fr), grape (gr), pungent (pn), sour (sr), sweet (sw), vinegar (vn), wine-like (wn), molasses (ml), color (cl), and browning (br).

Overall, berry the sweet, slightly sour and dark aromatics associated with ripe berries and grape (the sweet, fruity and musty aromatics commonly associate with grapes) were the most intense (mean score ca. 8) perceived attributes in Pj (Figure 6). Similar scores were observed for pungent (sharp, irritating physically penetrating sensation in the nasal cavity) and astringent (the dry puckering mouthfeel associated with an alum solution) in Pj4 and Pj18, while Pj21 was characterized by significantly (*P* < 0.05) lower values. Compared to Pj21, Pj4 and Pj18 showed a significantly (*P* < 0.05) higher sour taste, and a significantly (*P* < 0.05) lower sweet taste perception. Among the unfermented pomegranate juices, the attributes with the lowest values corresponded to anise, fermented, vinegar, wine-like and browening attributes (Figure 6). Fermentation caused in all the samples, the increase of the fermented, floral, and fruity intensity, and the decrease of the perceived grape, berry, sweet, and molasses attributes (Figure 6). Pungent score slightly decreased in FPj18 and FPj21 compared to corresponding unfermented controls, and sour perception increased in FPj18 and 21, but not in FPj4, this latter already characterized by an high value before fermentation. No significant (*P* > 0.05) differences were found between FPj and corresponding Pj in vinegar and wine-like attributes, and in color/browning intensity.

## Discussion

Oxidative stress-related diseases/disorders, i.e., metabolic, neurodegenerative, cardiovascular, mitochondrial diseases, and even cancer represent the most frequent causes of aging and death in the modern society (Singh et al., 2004; Halliwell, 2012; Chaturvedi and Beal, 2013). The intake of antioxidant compounds, from natural and synthetic sources, can control the magnitude of free radicals generation, preventing its undesirable effects, as well as support the organism antioxidant and detoxifying mechanisms (Yeh and Yen, 2006; Valko et al., 2007; Holst and Williamson, 2008; Kapravelou et al., 2015). Polyphenols are recognized as the most important natural antioxidant and anti-inflammatory agents provided by daily diet mainly through fruits and vegetables (Zhang and Tsao, 2016). However, to exert their biological properties these compounds need to be accessible to the organism and availability in the target tissue. Therefore, the investigation of the antioxidant activity of polyphenols should be supported by the evaluation of bioaccessibility and bioavailability (Carbonell-Capella et al., 2014). It has been largely reported that fermentation may further enhance the functional potential of vegetable materials through the metabolic activity of microorganisms involved, able to modulate the bioavailability of pre-existing functional compounds and to synthesize new ones (Filannino et al., 2013, 2018; Curiel et al., 2015). Lactic acid bacteria were successfully used for this purpose (Valero-Cases et al., 2017; Filannino et al., 2018). Recently, the fermentation with lactic acid bacteria of myrtle (*Myrtus communis* L.) and pomegranate juices have been investigated, finding relevant improvements of the antioxidant activity (Mousavi et al., 2011; Filannino et al., 2013; Curiel et al., 2015; Valero-Cases et al., 2017) and sensory properties (Filannino et al., 2013).

Aiming at investigating the potential effect of lactic acid bacteria to enhance the antioxidant activity, the juice from twenty accessions of pomegranate identified in Southern Italy (Puglia region) were fermented and characterized.

Pomegranate fruits were characterized before juice extraction. Overall, many of the characteristics of the pomegranate fruits of the local accessions (e.g., weight, sepal number, and sugar concentration) were similar with those of other biotypes previously identified in the Mediterranean area (Mars and Marrakchi, 1999; Martínez et al., 2006; Sarkosh et al., 2009; Tehranifar et al., 2010; Ferrara et al., 2011, 2014). Nevertheless, some accessions were characterized by yield of juice production higher than the widespread commercial cultivar Wonderful, included in the study as reference.

The juice was extracted from fresh arils and fermented with a selected strain of *L. plantarum*, a species characterized by large metabolic flexibility and good adaptation to different environments (Kleerebezem et al., 2003), included plant-derived matrices with high polyphenols concentration (Rodrìguez et al., 2009; Di Cagno et al., 2011; Filannino et al., 2013).

Nevertheless, since the pomegranate juice represents a hostile ecosystem (Filannino et al., 2013) for bacterial growth (e.g., pH 3.5 and high concentration of polyphenolic compounds), juices were supplemented with whey to get the optimal growth of the starter. Whey is considered as an agricultural surplus; moreover, it is characterized by a relevant organic load (high biological/chemical oxygen demand). From the environmental point of view, whey disposal is difficult, and pre-treatments are necessary (Prazeres et al., 2012). However, whey has a recognized nutritional value and its recycle into the food chain is desirable (Nath et al., 2016). In addition to proteins and lactose, whey also provides calcium, phosphorus, sulfur and water-soluble vitamins (Siso, 1996), and can be considered as a good supplement for the microbial growth (Bury et al., 1998). Fermentation lasted 24 h, allowing the growth of the starter to *ca.* 8.0 – 9.0 Log cfu/ml. Values of pH did not change significantly during the fermentation due to the buffering capacity of the pomegranate juice, even though a significant increase of organic acids concentration, and particularly lactic acid, was observed.

The antioxidant activity of Pj and FPj was first investigated through different *in vitro* analyses. Although already high before fermentation, the median values of scavenging activity of juices toward DPPH and ABTS radicals were significantly higher after fermentation. Valero-Cases et al. (2017) already reported increases of the *in vitro* antioxidant capacity in pomegranate juices fermented with lactic acid bacteria strains. Moreover, also the antioxidant capacity under simulated gastro-intestinal conditions increased mainly when *Lactobacillus* strains were used (Valero-Cases et al., 2017). Fermented juices FPj4, FPj18, and FPj21 showed the most intense antioxidant activity and the highest concentration of total phenols. The fermentation of plant materials leads to the increase of total phenolic compounds mainly due to the microbial hydrolysis reactions and biochemical changes of the products (acidic conditions). Hence, enhancing the bioactivity and digestibility (Rodrìguez et al., 2009; Hur et al., 2014). The influence of the degradation of phenolic acid esters and tannins by lactic acid bacteria esterase may also play a key role in the increasing of the antioxidant activity (Rodrìguez et al., 2009; Filannino et al., 2018). Several evidences on the tannase production and the release of ellagic acid during pomegranate juice fermentation by strains of *L. plantarum* were shown and speculated, respectively (Vaquero et al., 2004; Filannino et al., 2013). As previously reported for fermented myrtle juice (Curiel et al., 2015), pomegranate fermented juices were also able to inhibit the linoleic acid peroxidation during a long-time incubation. From a technological point of view, such feature can contribute to the long-term oxidative stability of foods and beverages in which the antioxidant matrix is included as ingredient (Shahidi and Zhong, 2010; Simic and Karel, 2013).

Oxidative stress and lipid peroxidation are responsible for the development of tissue damage and aging (Tuberoso et al., 2010; Gaschler and Stockwell, 2017). Aiming at verifying the activity of the fermented juices on a complex biological system, the protective ability toward oxidative stress was determined on mouse fibroblasts Balb3T3 cultures (Curiel et al., 2015).

Preliminarily, the cytotoxic effect of the juices was determined through the MTT assay, a routinely test employed to measure the cell viability. In detail, the cytotoxicity was only found at high concentrations (>50 mg/ml) of both unfermented and fermented juices. The low cytotoxicity excluded additional effects on mouse fibroblasts viability during the induction of the oxidative stress in presence of the pomegranate juices. Cells viability after oxidative treatments was determined by the MTT assay (Mosmann, 1983; Curiel et al., 2015). The protective effect of fermented juices on fibroblasts was similar to that of α-tocopherol, used as antioxidant reference, and higher than that of the unfermented ones. The intracellular ROS scavenging was also investigated on mouse fibroblasts by DCFH-DA assay. The results confirmed the significantly higher antioxidant activity of the fermented compared to unfermented juices, with FPj18 showing the most intense activity.

The phenolic profiles of Pj/FPj4, 18, and 21 were characterized aiming at identifying the changes induced by fermentation and responsible for the improved antioxidant activity. Delphinidin 3,5-diglucoside, cyanidin 3,5-diglucoside, delphinidin 3-glucoside, cyanidin–pentoside–hexoside, cyanidin 3-glucoside and pelargonidin 3-glucoside were found at similar concentration in both the samples. With the only exception of the cyanidin–pentoside–hexoside, which was reported in pomegranate for the first time, all the other compounds have previously been identified in this fruit (Hernádez et al., 1999; Fischer et al., 2011) at variable concentrations depending on cultivar (Alighourchi et al., 2008). Moreover, ellagitannins derivatives were also detected. Significant increases (up to *ca.* 60%) of HHDP-hexoside, ellagic acid hexoside and ellagic acid deoxy-hexoside occurred during fermentation, especially in FPj18. Ellagitannins, free ellagic acid and its derivatives deserve great interest from the food and beverage industry since considered multi-functional compounds with antioxidant, anti-inflammatory, antitumoral, antiviral, antimicrobial, and hypocholesterolemic activities (Fukuda et al., 2003; Ascacio-Valdés et al., 2011; Fracassetti et al., 2013).

The hydrolysis of ellagitannins through tannases (Khanbabaee and van Ree, 2001) leads to the release of the unstable HHDP (hexahydroxydiphenoyl acid) unit. A lactonization generally causes the conversion of HHDP into a stable form, which acts as precursor for gallic, ellagic, quinic, caffeic, and ferulic acids, all characterized by high antioxidant activity (Ascacio-Valdés et al., 2011). Vaquero et al. (2004) investigated tannase activity of lactobacilli belonging to the genera of *Lactobacillus*, *Leuconostoc*, *Oenococcus*, and *Pediococcus*. A tannase has already been identified in *L. plantarum*, and characterized (Iwamoto et al., 2008; Curiel et al., 2009; Ren et al., 2013). The presence of tannases in *L. plantarum* strains suggested a specific catabolic capacity of this species against tannins, probably a response mechanism to overcome the inhibitory effect of such compounds (Jiménez et al., 2014). Moreover, the presence of a second active extracellular tannase (Jiménez et al., 2014) could provide them the additional capability to degrade tannins unable to pass the membrane. Moreover, degradation of polyphenol and tannins into aglycones and, occasionally, various simple aromatic acids can also occur during human digestion through the activity of oral and intestinal microorganisms (Petti and Scully, 2009; Viuda-Martos et al., 2010). Metabolization of the ellagitannins by the colonic microflora into bioavailable urolithins (hydroxy-6H-dibenzopyran-6-one derivatives) was showed in healthy subjects consuming pomegranate juice daily for 5 days (Cerdá et al., 2004).

In agreement with previous findings (Filannino et al., 2013; Di Cagno et al., 2017) the sensory analysis of the juices confirmed the suitability of the fermentation to improve the floral/fruity perceptions and the attenuation of the astringent/pungent attributes, without increase of the vinegar/wine-like notes.

## Conclusion

Lactic acid bacteria fermentation was already recognized as effective in improving sensory properties and the shelf-life of fruit juices. Although further research is needed to evaluate the bioaccessibility and bioavailability of the phenolic compounds, this study demonstrated that fermentation with *L. plantarum* also improved the antioxidant activity of pomegranate juice, thus hypothesizing its use as functional beverage or ingredient for novel functional formulations. Moreover, *in vitro* and *ex vivo* analyses showed that, among the twenty pomegranate local accessions included in the study, the fermented juices from Bitonto Piscina (Pj4) and Sanrà nero (Pj18) had antioxidant activity higher than the reference Wonderful cultivar and can be considered as suitable candidates for breeding and large-scale cultivation.

## Data Availability

The raw data supporting the conclusions of this manuscript will be made available by the authors, without undue reservation, to any qualified researcher.

## Author Contributions

EP elaborated the results of juice fermentation and *in vitro* antioxidant activity analysis and wrote the final draft of the manuscript. MM carried out the pomegranate juice fermentation and *in vitro* antioxidant activity analysis. DP carried out the experiments of antioxidant activity on biological system. BM elaborated data of antioxidant activity on biological system. AT carried out the experiments of phenolic compounds profile. GF elaborated data of pomegranate fruits and juices characterization, and critically revised the manuscript. AM carried out the fruit harvesting and characterization. MG critically revised the manuscript. CR conceived the study, coordinated the work, and critically revised the manuscript. All authors read and approved the final version of the manuscript.

## Conflict of Interest Statement

DP and BM were employed by the Giuliani S.p.A., Italy. The remaining authors declare that the research was conducted in the absence of any commercial or financial relationships that could be construed as a potential conflict of interest.
