# Supplementary material for: Lactic Acid Fermentation of Pomegranate Juice as a Tool to Improve Antioxidant Activity
Source: Front Microbiol. 2019 Jul 3;10:1550. doi: 10.3389/fmicb.2019.01550 (PMC6619386; doi:10.3389/fmicb.2019.01550)
Supplement: Supplementary file 1 [file Table_1.DOCX]

**Supplementary Table S1**. Morphological characteristics of the pomegranate fruits.

| Accession | Code | Fruit weight  (g.) | Fruit volume  (cm^3^) | Fruit diameter  (mm) | Fruit length  (mm) | Sepals  (n.) | Calyx diameter  (mm) | Calyx length  (mm) |
| --- | --- | --- | --- | --- | --- | --- | --- | --- |
| Acido Torrelonga | 1 | 365.7^cd^ | 350^cd^ | 92.5^b^ | 94.9^ab^ | 6.8^ab^ | 27.4^a^ | 14.3^abc^ |
| Bariblu | 2 | 181.5^fgh^ | 150^fg^ | 73.6^def^ | 77.0^cdefg^ | 6.0^bc^ | 21.0^bc^ | 12.7^abcde^ |
| Bitetto dolce | 3 | 288.4^def^ | 300^cde^ | 81.8^cde^ | 70.4^defgh^ | 6.5^b^ | 20.6^bcd^ | 15.5^a^ |
| Bitonto Piscina | 4 | 115.5^h^ | 100^g^ | 62.2^g^ | 64.8^fgh^ | 6.0^bc^ | 15.1^fg^ | 10.4^de^ |
| Bitonto T | 5 | 314.4^cde^ | 300^cde^ | 87.5^bc^ | 94.4^ab^ | 6.0^bc^ | 15.3^efg^ | 12.1^abcde^ |
| Campus | 6 | 265.9^defg^ | 225^efg^ | 83.0^cd^ | 82.1^cd^ | 6.0^bc^ | 15.6^defg^ | 12.9^abcde^ |
| Demarco | 7 | 161.0^h^ | 170^efg^ | 70.4^fg^ | 63.8^gh^ | 4.5^c^ | 12.3^g^ | 14.7^ab^ |
| Dolce Conversano | 8 | 394.3^c^ | 375^c^ | 94.0^b^ | 97.0^ab^ | 6.8^ab^ | 19.7^cdef^ | 12.8^abcde^ |
| Dolce Corallo | 9 | 223.1^efgh^ | 225^efg^ | 78.7^def^ | 77.7^cdef^ | 6.3^b^ | 18.9^cdef^ | 14.0^abcd^ |
| Funno Dolce | 10 | 267.8^defg^ | 200^efg^ | 82.1^cde^ | 88.7^bc^ | 6.8^ab^ | 15.4^efg^ | 11.8^abcde^ |
| Japigia B | 11 | 180.8^fgh^ | 188^efg^ | 73.8^def^ | 76.7^cdefg^ | 6.8^ab^ | 16.8^cdefg^ | 10.7^cde^ |
| Acido Capurso | 12 | 548.0^b^ | 525^b^ | 105.4^a^ | 102.2^a^ | 8.0^a^ | 24.0^b^ | 15.4^a^ |
| Macello Triggiano | 13 | 197.6^gh^ | 225^efg^ | 77.2^cdef^ | 74.8^cdefgh^ | 5.5^bc^ | 18.0^cdef^ | 11.5^bcde^ |
| Melograno Foggia | 14 | 268.3^defg^ | 238^def^ | 84.3^cd^ | 78.6^cdef^ | 6.8^ab^ | 14.9^fg^ | 10.0^e^ |
| Molfetta Acido | 15 | 217.6^efgh^ | 200^efg^ | 79.0^cdef^ | 74.4^defgh^ | 6.5^b^ | 20.5^bcde^ | 11.7^bcde^ |
| Mungivacca Tardivo | 16 | 235.5^efgh^ | 250^def^ | 82.8^cd^ | 81.0^cde^ | 6.0^bc^ | 18.5^cdef^ | 12.6^abcde^ |
| Ottantara | 17 | 180.5^fgh^ | 175^efg^ | 72.0^efg^ | 78.3^cdef^ | 6.3^b^ | 19.6^cdef^ | 10.9^bcde^ |
| Sanrà nero | 18 | 283.6^defg^ | 250^def^ | 82.8^cd^ | 94.2^ab^ | 6.3^b^ | 16.9^cdefg^ | 10.9^bcde^ |
| Modugno | 19 | 168.1^fgh^ | 150^fg^ | 71.8^efg^ | 66.9^efgh^ | 5.3^bc^ | 17.5^cdef^ | 11.0^bcde^ |
| Triggiano | 20 | 200.3^efgh^ | 213^efg^ | 71.6^efg^ | 62.8^h^ | 6.3^b^ | 16.1^cdefg^ | 14.3^abc^ |
| *Mean value* |  | *252.9 ± 42.6* | *240 ± 45.2* | *80.3 ± 3.8* | *80.0 ± 5.0* | *6.3 ± 0.5* | *18.2 ± 1.8* | *12.5 ± 1.2* |
| Wonderful* | 21 | 616.1^a^ | 648^a^ | 100.0^a^ | 87.2^b,c^ | 6.0^b,c^ | 24.9^b^ | - 1. ^abcde^ |

*Wonderful was considered as the reference cultivar.

Different letters within a column refer to a difference significant at P ≤ 0.05, as obtained by the REGWQ test.
